# Feasibility of inguinal hernioplasty under local anaesthesia in elderly patients

**DOI:** 10.1186/1471-2482-12-S1-S2

**Published:** 2012-11-15

**Authors:** Bruno Amato, Rita Compagna, Gianni Antonio Della Corte, Giovanni Martino, Tommaso Bianco, Guido Coretti, Roberto Rossi, Francesca Fappiano, Giovanni Aprea, Alessandro Puzziello

**Affiliations:** 1 Department of General, Geriatric, Oncologic Surgery and Advanced Technologies, University of Naples Federico II, Via S. Pansini,5 – 801311 Napoli, Italy; 2 Departmet of Medical and Surgical Sciences, University Magna Graecia, Catanzaro, Italy

## Abstract

**Background:**

The aim of this study is to evaluate the feasibility and the safety of hernioplasty under local anaesthesia in elderly patients with significant comorbidity.

**Methods:**

A total of 218 patients underwent inguinal hernia repair with mesh between June 2009 and July 2012. Presence of comorbid conditions and complications were compared between patients younger and older than 70 years.

**Results:**

Hernia repair in older patients were more likely associated with comorbid conditions than in their younger counterparts ( hypertension: 25% vs 8.16%; cardiovascular diseases: 50% vs 22%; benign prostatic hypertrophy 60% vs 30%). The most common postoperative complications in both groups were recurrence, wound infection, urinary retention. There was a slightly higher rate of complication in elderly group.

**Conclusions:**

Inguinal hernia repair with local anaesthesia is quite safe and results in a good success rate in elderly patients despite a higher rate of comorbidity typical of this kind of patient.

## Background

Inguinal hernia is a very common disease and it is more frequent in elderly than in younger patients. The incidence rises from 11 per 10,000 person-years aged 16-24 years to 200 per 10,000 person-years aged 75 years and above [1]. Inguinal hernia repair is one of the most commonly performed operations worldwide [2]. Moreover the demand for surgical services for inguinal hernia is increasing due to an increase of an ageing population [3]. This pathology causes aching discomfort and an unsightly swelling and it is established that hernioplasty gives patients a remarkable improvement of quality of life also in geriatrics [3-5]. In addition, a routine elective operation allow us to avoid acute complications like strangulation, intestinal obstruction, and infarction that are the most important complications of untreated hernia, and are potentially life-threatening [6].

According to the literature the safest approach to geriatric patients is to perform hernioplasty in open surgery [7] using local anaesthesia [8,9] but some authors suggests that it is better to take a watchful waiting approach especially in asymptomatic patients [10,11]. In our study we evaluate the impact of hernioplasty in local anesthesia in patients younger and older than 70 years. We compared the frequency of complications and comorbidity in the two groups in order to verify the feasibility and safety of this approach in elderly.

## Methods

In the study we included patients with inguinal hernia that reached our department in the period between June 2009 and July 2012. Patients with recurrence, bilateral hernia, large size hernia and all cases that underwent general anaesthesia were excluded from the study. A total of 218 hernioplasties were performed by resident surgeons in local anaesthesia, 98 of these were patients under 70 years and 120 patients were over this age. All procedures were performed as described by Rutkow and Robbins [12] or with Lichtenstein techniques with standard-weight polypropylene mesh [13]. Prophylactic antibiotics were not used [14]. American Society of Anaesthesiologists (ASA) grade 1 and 2 patients underwent deep sedation (midazolam, fentanyl) combined with a field block of local anaesthesia (lidocaine without adrenaline) and were monitored by pulse oximetry. For ASA grade 3 and 4 patients procedures were performed in the presence of the anaesthetist and deep sedation was not used [9,15,16]. Comorbidity was registered in the pre-operative time. After surgery patients were examined after 1 week and a second time after 1 month in order to evaluate the presence of post-operative complications.

## Results

None of the 218 procedures were complicated by any side-effects due to the anesthetic techniques or any other procedure-related complication and there were no deaths. There was a higher rate of comorbidity in the group of over 70 years of age. The main associated illnesses that were observed are: benign prostatic hypertrophy (BPH 60% in over 70 and 30% in under 70), cardiovascular diseases ( CD 50% in over 70 and 22% in under 70), hypertension ( 25% in over 70 and 8.16% in under 70), chronic obstructive pulmonary disease (COPD; 12.5% in over 70 and 6.12% in under 70). Other comorbidities that we found were obesity, diabetes, hepatic cirrhosis and renal failure [ Table 1]. Intraoperative complications that occurred were severe bleeding (5 cases) and arrhythmia (3 cases). Post-operative complications that occurred were: recurrence (12 cases), wound infection (11 cases),nerve entrapment (10 cases), mesh infection (6 cases), urinary retention (5 cases), and wound hematoma (4 cases). There was a slightly higher rate of complications in the elderly group, in fact recurrence occurred in 7.5% of older versus 3.6%, wound infections 6.6% versus 3.6%, mesh infection in 3.3% versus 2.04%, urinary retention in 3.3% versus 1.02% [Table 2]. Overall the complication rate is not particularly high even in patients who have a higher percentage of comorbidity.

**Table 1:** Patients characteristics

N = 218		
AGE	Under 70 98 ( 45% )	Over 70 120 ( 55%)

ASA 1 -2	124 ( 76%)	39 (23%)
ASA 3-4	8 (14.5%)	47 (85.4%)
OBESITY	15 (15.3%)	8 (7%)
HYPERTENSION	8 (8.16%)	30 ( 25%)
CD	22 (22%)	60 (50%)
DIABETES	5 (5.1%)	12 (10%)
BPH	30 (30%)	72 (60%)
CIRRHOSIS / HEPATITIS	2 ( 2.04%)	11 (9%)
RENAL FAILURE	1 (1.04%)	4 (3.3%)
COPD	6 (15%)	15 ( 12.5%)

**Table 2:** Complications

N = 218		
AGE	Under 70 98 ( 45% )	Over 70 120 ( 55%)

SEVERE BLEEDING	1 ( 1.02%)	4 (3.3%)
ARRHYTHMIA	0	3 (2.5%)
RECURRENCE	3 (3.6%)	9 (7.5%)
WOUND HEMATOMA	1 (1.02%)	3 ( 2.5%)
WOUND INFECTION	3 (3.6%)	8 (6.6%)
URINARY RETENTION	1 (1.02%)	4 (3.3%)
MESH INFECTION	2 (2.04%)	4 (3.3%)
NERVE ENTRAPMENT	2 ( 2.04%)	8 (6.6%)

## Conclusions

Elective inguinal hernia repair under local anesthetic has a good outcome also in the elderly even if there are significant comorbidities. The slightly higher rate of complications that occurred in older patients is not significant and does not support advising against the use of this surgical approach in elderly. So in our opinion hernioplasty under local anesthesia is quite safe and feasible also in patients over 70 years.

## List of abbreviations

ASA: American Society of Anaesthesiologists; BPH: benign prostatic hypertrophy; CD: cardiovascular disease; COPD: chronic obstructive pulmonary disease.

## Competing interests

The authors declare that they have no competing interests.

## Authors' contributions

BA: conception and design, interpretation of data, given final approval of the version to be published; RC: acquisition of data, drafting the manuscript, given final approval of the version to be published; ADC: acquisition of data, drafting the manuscript, given final approval of the version to be published; GM: acquisition of data, drafting the manuscript, given final approval of the version to be published; TB: acquisition of data, drafting the manuscript, given final approval of the version to be published; GC: acquisition of data, drafting the manuscript, given final approval of the version to be published; RR: acquisition of data, drafting the manuscript, given final approval of the version to be published; FF: acquisition of data, drafting the manuscript, given final approval of the version to be published; GA, AP: conception and design, given final approval of the version to be published.
